# Multimodal Imaging and Clinical Implications of Collagenous Fibroma in the Juxtaforaminal Premaxillary Fat Pad Mimicking Locoregional Tumor Recurrence: A Case Report and Literature Review

**DOI:** 10.2174/0115734056391917250730080108

**Published:** 2025-08-04

**Authors:** Jeong Pyo Lee, Hye Jin Baek, Ki-Jong Park, Jin Pyeong Kim, Hyo Jung An, Eun Cho

**Affiliations:** 1 Department of Radiology, Jinju Good Morning Hospital, 213 Beon-gil, 7 Seojangdae-ro, Jinju 52653, Republic of Korea; £ Present Address: Department of Radiology, Gyeongsang National University Hospital, 79 Gangnam-ro, Jinju 52727, Republic of Korea; 2 Miracle Radiology Clinic, 201, Songpa-daero, 05854 Songpa-gu, Seoul, Republic of Korea; £ Present Address: Department of Radiology and Research Institute for Convergence of Biomedical Science and Technology, Pusan National University Yangsan Hospital, Pusan National University School of Medicine, 20 Geumo-ro, Mulgeum-eup, Yangsan-si, Gyeongsangnam-do 50612, Republic of Korea; 3 Department of Neurology, Jinju Good Morning Hospital, 213 Beon-gil, 7 Seojangdae-ro, Jinju 52653, Republic of Korea; 4 Department of Otorhinolaryngology-Head and Neck Surgery, Gyeongsang National University School of Medicine and Gyeongsang National University Changwon Hospital, 11 Samjeongja-ro, Seongsan-gu, Changwon 51472, Republic of Korea; 5 Department of Pathology, Gyeongsang National University School of Medicine and Gyeongsang National University Changwon Hospital, 11 Samjeongja-ro, Seongsan-gu, Changwon 51472, Republic of Korea; 6 Department of Radiology, Gyeongsang National University School of Medicine and Gyeongsang National University Changwon Hospital, 11 Samjeongja-ro, Seongsan-gu, Changwon 51472, Republic of Korea

**Keywords:** Collagenous fibroma, Desmoplastic fibroblastoma, Soft tissue tumor, CT, MRI, Ultrasonography, Adenoid cystic carcinoma

## Abstract

**Background:**

Collagenous fibroma (CF), or desmoplastic fibroblastoma, is a rare benign tumor with few reported cases involving the facial region. Its presence in uncommon sites can pose diagnostic challenges due to overlapping clinical and radiologic features with malignant neoplasms.

**Case Presentation:**

Herein, we report a case of a 48-year-old female with CF in the juxtaforaminal premaxillary fat pad, presenting with neuralgic pain extending to the ipsilateral upper gingiva. The patient had a history of adenoid cystic carcinoma (AdCC) of the right nasolabial fold, which was treated surgically four years prior. During evaluation with a multimodal radiologic approach using ultrasonography, CT, and MRI, the lesion was revealed to be a soft tissue lesion in the premaxillary region, raising suspicion of recurrent AdCC. However, histopathologic examination of the surgical excision confirmed the diagnosis of CF.

**Conclusion:**

This case highlights the importance of integrating clinical history, imaging findings, and pathological analysis for accurate diagnosis and appropriate management.

## 1 INTRODUCTION

Collagenous fibroma (CF), also known as desmoplastic fibroblastoma, is a rare, benign soft tissue tumor first described by Evans in 1995 [1]. It is characterized by a paucicellular matrix of spindle or stellate fibroblasts within a dense collagenous stroma and typically affects the subcutaneous or deep soft tissues of middle-aged adults [2]. This tumor predominantly arises in the subcutaneous or intramuscular regions of the extremities, joints, and neck [3]. There are few reports in the literature regarding CF involving the face; however, the reported cases have shown limited information, owing to the limited availability of imaging modalities and the overall poor quality of representative images. Here, we present a case of a 48-year-old female with CF located in the right premaxillary fat pad near the infraorbital foramen. The patient had a history of adenoid cystic carcinoma (AdCC) in the right nasolabial fold, which was surgically treated four years earlier. To the best of our knowledge, this is the first report of CF arising from the premaxillary region, accompanied by high-quality multimodal radiologic images. This report emphasizes that CF, although a benign entity, can mimic malignancies, particularly in patients with a prior history of cancer, and also highlights the importance of a multidisciplinary evaluation in managing such challenging scenarios.

## 2 CASE REPORT

**Fig. (1):**
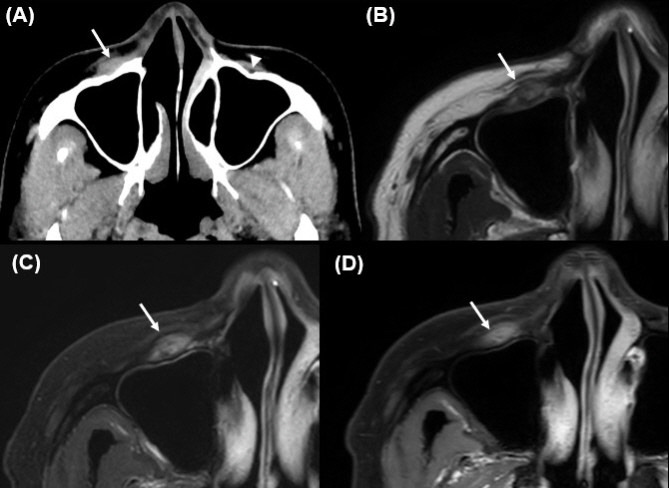
Cross-sectional images, including CT and MRI. (**A**) Contrast-enhanced axial CT image shows a mildly enhancing focal soft tissue lesion (arrow) in the right premaxillary fat pad, involving the ipsilateral infraorbital foramen (arrowhead; contralateral side shown for comparison). (**B**–**D**) Axial MR images demonstrate the lesion as mixed low to isointensity on T1-weighted imaging (**B**), inhomogeneous hyperintensity with focal linear hypointense foci on fat-suppressed T2-weighted imaging (**C**), and mild to moderate heterogeneous enhancement relative to adjacent muscle and fat planes on contrast-enhanced fat-suppressed T1-weighted imaging (**D**). The lesion extends into the juxtaforaminal fat pad and partially involves the levator labii superioris muscle. These findings, including proximity to and suspected involvement of the infraorbital nerve, raised concern for locoregional tumor recurrence in a patient with prior adenoid cystic carcinoma.

**Fig. (2):**
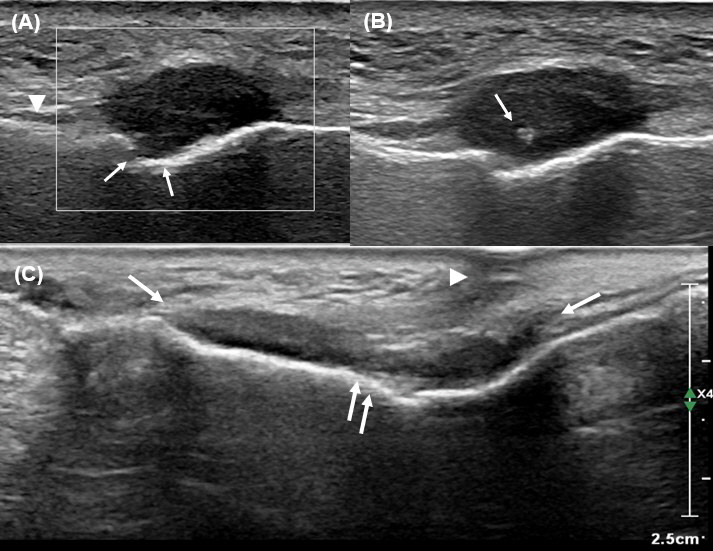
Face ultrasonography (US). (**A**) Longitudinal US image shows a focal hypoechoic nodular lesion in the right premaxillary fat pad, involving the levator labii superioris muscle (arrowhead) and abutting the infraorbital nerve branch (arrows). No internal vascularity is seen on Doppler, suggestive of a non-vascular soft tissue lesion. (**B**) US-guided fine needle aspiration biopsy was performed during the same session (needle tip: arrow), targeting the hypoechoic component. (**C**) On follow-up US performed 5 months later, the lesion shows interval growth in both size and extent (arrows), with newly developed overlying skin retraction and hypoechoic changes in the subcutaneous fat layer (arrowhead), suggesting progression with superficial extension.

**Fig. (3):**
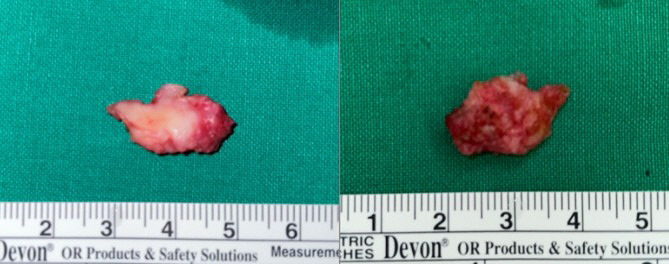
The lesion was surgically excised *via* a transoral Caldwell-Luc approach. The resected specimen revealed a firm, multilobulated, whitish nodular mass measuring up to 2.5 cm in maximum diameter. The lobulated and fibrous appearance corresponded with the collagenous nature of the tumor.

**Fig. (4):**
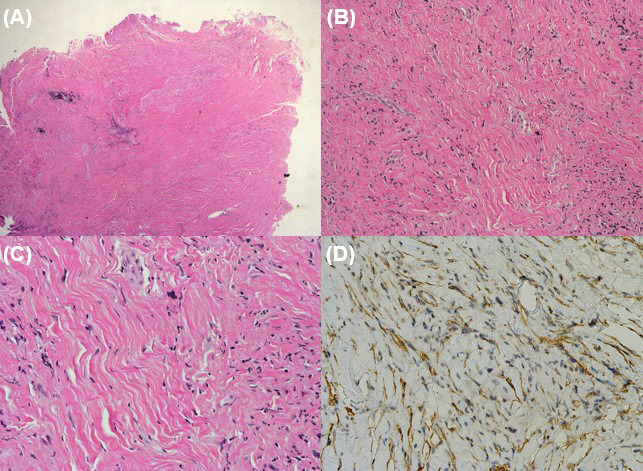
Histopathologic results. (**A**) Low-power view (×10, hematoxylin and eosin stain) shows an ill-defined, hypocellular soft tissue lesion without necrosis. (**B**) Higher magnification (×100) reveals bland spindle cells distributed within a densely collagenous stroma. (**C**) At higher power (×200), the lesion is composed of stellate fibroblasts without nuclear pleomorphism or mitotic activity. Multifocal lymphocytic infiltration and focal proliferation of peripheral nerve fascicles are also noted, suggestive of reactive neurogenic changes. (**D**) Immunohistochemically, the spindle cells show focal positivity for smooth muscle actin (SMA) (×200), supporting a myofibroblastic origin.

This was an observational case study that did not alter the patient’s management and clinical outcomes. Therefore, ethical approval was not required for this case report, and patient consent was waived due to the retrospective nature of the study. A 48-year-old female presented with a palpable lesion in the right cheek, associated with neuralgic pain radiating to the right upper gingiva for the past 2 months. The patient had undergone surgical resection for AdCC of the right nasolabial fold four years prior. No other significant medical or surgical history was noted. Physical examination revealed a firm and non-movable lesion, measuring 2.0 cm, in the subcutaneous layer of the right cheek. The patient complained of neuralgic pain during palpation of the lesion. No overlying skin changes or mucosal abnormalities were observed. Routine laboratory tests were also normal. CT images revealed a mildly enhancing focal soft tissue lesion in the right premaxillary fat pad at the site of palpation (Fig. **1A**). On MRI, the lesion in the right premaxillary fat pad exhibited mildly inhomogeneous enhancement, appearing as mixed low to isointense signals on T1-weighted images and showing inhomogeneous hyperintensity with focal linear hypointense foci on T2-weighted images (1.7 cm × 1.0 cm × 0.6 cm) (Fig. **1B-D**). Additionally, the lesion involved the levator labii superioris muscle and was located in the juxtaforaminal fat pad near the infraorbital foramen (Fig. **1B-D**). These findings raised suspicion for recurrent AdCC, and we also considered tumefactive fibroinflammatory lesions, such as inflammatory pseudotumor or IgG4-related disease, as a differential diagnosis. To further evaluate the lesion and obtain tissue samples, ultrasonography (US) was performed using a 5 to 12 MHz linear-array transducer. US images revealed a partly defined, focal hypoechoic nodular lesion without definite intralesional vascularity in the right premaxillary fat pad, located near the previous surgical site (Fig. **2A**). During the same session, a US-guided fine-needle aspiration (FNA) was also performed once after local anesthesia with 1% lidocaine (Fig. **2B**). The procedure was uneventful. However, the patient experienced severe neuralgic pain. On cytologic examination, numerous inflammatory cells, including neutrophils, were observed, with no evidence of malignancy. Based on the cytologic results, the clinician opted for short-term regular follow-up rather than surgical excision. At a 5-month follow-up, the patient complained of interval aggravation of the neuralgic pain. Follow-up US images showed an increase in size and extent of the lesion, measuring up to 2.3 cm in maximum diameter, with newly developed focal skin retraction overlying the inferior portion of the lesion (Fig. **2C**). Consequently, the lesion was excised *via* a Caldwell-Luc operation, and the lesion was a multilobulated whitish nodular lesion up to 2.5 cm in maximum diameter (Fig. **3**). The frozen section of the mass revealed a whitish soft tissue nodule measuring 2.0 cm × 1.0 cm × 0.8 cm. Histopathological examination showed an ill-defined, hypocellular tumorous lesion without any necrotic areas (×10 hematoxylin and eosin) (Fig. **4A**). At higher magnification, bland-looking spindle cells were interspersed within a dense hypovascular collagenous stroma (×100 hematoxylin and eosin) (Fig. **4B**). The spindle cells consisted of stellate cells and fibroblasts with a patternless distribution; however, there was no evidence of nuclear pleomorphism or mitosis. Additionally, multifocal lymphocytic infiltrates and multifocal proliferations of nerve fascicles were noted (×200, hematoxylin and eosin) (Fig. **4C**). Immunohistochemical staining was positive for SMA (Fig. **4D**), desmin, S-100, and LCA, but negative for ALK1, beta-Catenin, CD34, cytokeratin, and EMA. Based on these results, the lesion was confirmed to be CF. Postoperative recovery was uneventful, and the patient's neuralgic symptoms were significantly resolved. Over 3.5 years of follow-up, no recurrence or residual symptoms were noted. Although molecular alterations, such as FOSL1 gene rearrangement and t(2;11)(q31;q12) translocation, have been identified in some cases of collagenous fibroma, these were not assessed in our case, as the diagnosis was established based on typical histologic and immunohistochemical findings.

## 3 DISCUSSION

CF is a rare, benign tumor that primarily affects the deep soft tissues of the trunk and extremities, although sporadic cases of involvement in the head and neck have been reported [4] (Table **1**). However, facial CF is exceptionally rare, with very few documented cases in the facial region. To the best of our knowledge, this is the first case to integrate ultrasonography, CT, and MRI findings as a multimodal imaging approach in the diagnostic process for CF in the juxtaforaminal premaxillary fat pad, which mimicked locoregional tumor recurrence in a patient with AdCC. Only a few prior reports have described CF in the facial region, and most lacked detailed imaging analysis or high-resolution images. For example, Liu *et al*. [5] presented a nasal CF case with nonspecific MRI findings, while Yamamoto *et al*. [6] described CF with rim enhancement on post-contrast T1-weighted images in musculoskeletal sites. However, none of the prior studies have incorporated a multimodal approach integrating US, CT, and MRI in a single case. Thus, this case offers a unique contribution to the radiologic characterization of CF in the head and neck region.

Preoperative radiological diagnosis is important to prevent overtreatment and unnecessary extensive procedures, particularly in patients with a history of malignancy. However, the imaging characteristics of CF remain poorly established due to its rarity and the predominance of reported cases in the extremities. Consequently, generalizing the imaging findings of CF is still challenging.

On CT, CF typically appears as a nonspecific, inhomogeneous soft tissue density mass. It generally demonstrates low to intermediate attenuation, with areas of soft tissue density visible on unenhanced scans. On contrast-enhanced CT, heterogeneous enhancement may be observed due to the fibrous nature of the tumor, although this can vary depending on the degree of fibrosis and vascularity [7]. MR findings of CF are variable, typically showing low to intermediate signal intensity on T1-weighted images, appearing slightly hypointense compared to adjacent muscle. On T2-weighted imaging, the lesion appears inhomogeneous with hyperintensity interspersed by low-signal areas, which are more hyperintense than muscle but less intense than adjacent fat, likely reflecting a dense fibrous stroma. Post-contrast T1-weighted images often reveal rim or heterogeneous enhancement. Minimal internal enhancement may indicate reduced vascularity, as collagen fibers can restrict contrast agent diffusion, while rim enhancement suggests the presence of abundant peripheral blood vessels [5, 6]. In contrast to musculoskeletal CFs, which occasionally show rim enhancement [6], our lesion did not demonstrate a defined peripheral rim, possibly due to the anatomical differences and lower tissue contrast in the facial region.

In the present case, the lesion exhibited inhomogeneous T2 hyperintensity with a few linear T2 hypointense foci. Based on the histopathologic findings, the main T2 hyperintense areas corresponded to hypercellular regions containing numerous inflammatory cells (primarily neutrophils) with relatively fewer collagen fibers, whereas the focal T2 hypointense foci represented hypocellular regions with dense collagen fibers. The lesion demonstrated inhomogeneous mild enhancement; however, most areas showed enhancement. This enhancement pattern is likely influenced by tissue composition, including the amount of collagen fibers and cellularity [8, 9]. Previous studies have reported that hypercellular areas and regions with expanded extracellular space (dense collagen) increase the volume of distribution and slow washout kinetics, allowing greater gadolinium accumulation [10, 11].

In clinical practice, most soft tissue tumors typically exhibit high signal intensity on T2-weighted images. However, CF may demonstrate low T2 signal intensity due to its low cellularity and high collagen content [12]. Nevertheless, low T2 signal intensity is not specific to CF and can also be observed in various other soft tissue masses, such as aggressive fibromatosis, neurofibroma, malignant fibrous histiocytoma, and masses with calcification [13]. While soft tissue tumors, such as aggressive fibromatosis, neurofibroma, and malignant fibrous histiocytoma, may demonstrate overlapping imaging features, such as low T2 signal intensity or mild enhancement, they often exhibit additional characteristic findings, including the fascial tail sign, target-like appearance, or infiltrative margins with necrotic components. In contrast, CF lacks such secondary diagnostic features, which makes its imaging presentation relatively nonspecific. Furthermore, as CF does not exhibit specific imaging characteristics even in previously reported musculoskeletal cases, and given the extreme rarity of facial involvement, the diagnosis may be easily overlooked. Thus, CF should be considered as a possible, albeit uncommon, differential diagnosis when evaluating atypical subcutaneous facial lesions or facial neuralgia in patients with a history of malignancy. Given the nonspecific imaging features of CF, even on delayed-enhanced MRI and other advanced modalities, accurate preoperative diagnosis is often difficult. Therefore, histopathologic confirmation remains essential.

In addition, our patient experienced neuralgic pain in the right cheek, radiating to the ipsilateral upper gingiva. Based on radiological findings during the work-up, we hypothesized that this pain originated from direct involvement of the infraorbital nerve, a branch of the maxillary division of the trigeminal nerve, by the lesion. However, histopathologic examination of the surgical specimen revealed no direct tumor infiltration of the nerve. Instead, CF with multiple inflammatory cells encased the nerve fascicles, and multifocal proliferation of nerve fascicles was observed. These findings suggest that the neuralgic pain may have been triggered by the reactive hypertrophy of the nerve, rather than by direct tumor invasion.

Furthermore, most existing literature on CF originates from musculoskeletal imaging of lesions in the trunk or limbs. Therefore, applying diagnostic frameworks based on musculoskeletal CF to head and neck lesions is not appropriate, given the anatomical complexity and differing spectrum of common lesions in the HN region. In particular, CF does not demonstrate uniquely pathognomonic imaging features, further complicating differential diagnosis. When encountering a rare lesion in the premaxillary or facial region, differential considerations should focus on entities that are more frequently encountered in this location, such as sinonasal lymphoma, squamous cell carcinoma, minor salivary gland tumor, and tumefactive disease, such as IgG4-related disease. This consideration was reflected in our diagnostic approach, especially given the patient’s history of AdCC.

In addition, surgical resection was performed due to progressive infraorbital neuralgia and interval growth of the lesion observed on follow-up imaging. Given the lesion’s juxtaforaminal location and proximity to the infraorbital nerve, wide local excision was performed to ensure complete removal and symptomatic relief. In most reported cases, surgical excision is considered curative, with low recurrence rates documented in the literature [10, 14]. The patient underwent imaging follow-up every 6 months for 3.5 years postoperatively, with no recurrence observed during that time. While the prognosis of collagenous fibroma is generally excellent, certain factors may influence recurrence risk, including incomplete resection, lesion proximity to critical neurovascular structures, or atypical histologic features, such as hypercellularity or mitotic activity [1, 10]. In our case, none of these factors were identified, and the patient remained recurrence-free over 3.5 years of surveillance.

Through this report, we also emphasize the diagnostic challenge posed by postoperative lesions in cancer survivors. As this is only the second reported case of CF occurring near a surgical site [14], it underscores the diagnostic challenge posed by the overlapping clinical symptoms and radiologic features of benign and malignant entities. In such cases where recurrence is suspected, a tissue biopsy is essential whenever feasible. In our case, preoperative cytologic results were negative for malignancy (showing only many inflammatory cells). However, the possibility of recurrent AdCC could not be entirely ruled out. During the follow-up period, our patient experienced worsening neuralgic pain, and the lesion showed radiologic progression. As demonstrated in our case, regular short-interval radiologic follow-up is a valuable strategy among the available options for monitoring lesion changes, determining the optimal timing for surgery, ensuring an accurate diagnosis, and planning future treatment strategies. Therefore, obtaining an accurate tissue diagnosis is essential not only to confirm the nature of the lesion but also to guide appropriate treatment decisions and ensure favorable patient outcomes.

## CONCLUSION

This case highlights the diagnostic complexities of CF in patients with a history of malignancy. Accurate diagnosis relies on a combination of clinical, radiologic, and histopathologic evaluations. Timely diagnosing of this rare tumor is crucial to avoid unnecessary aggressive interventions and ensure appropriate patient management.

## AUTHORS’ CONTRIBUTIONS

The authors confirm their contribution to the paper as follows: H.J.B. and K.J.P.: Conceptualization; J.P.L. and J.P.K.: Methodology; H.J.B., H.J.A., and E.C.: Analysis and interpretation of results; J.P.L., J.P.K., and E.C.: Validation; J.P.L., H.J.B., and K.J.P.: Draft manuscript. All authors reviewed the results and approved the final version of the manuscript.

## Figures and Tables

**Table 1:** Reported cases of collagenous fibroma with imaging features.

**First Author (Year)/ Refs.**	**Location**	**Imaging Modalities**	**Key Features**
Evans [1]	Various (mostly extremities)	None reported	Initial pathologic characterization only
Walker *et al*. [3]	Shoulder	CT	Well-defined soft tissue mass
Liu *et al*. [5]	Nasal cavity	CT, MRI	Heterogeneous enhancement, soft tissue mass
Yamamoto *et al*. [6]	Shoulder, neck	MRI	Rim enhancement on postcontrast T1WI
Giuffrida *et al*. [10]	Various	None reported	Pathologic focus, no imaging
Nishio *et al*. [11]	Various	None reported	Genetic analysis; no imaging
Shuto *et al*. [13]	Shoulder	CT, MRI	Soft tissue mass with enhancement
Kim *et al*. [14]	Post-thyroidectomy neck	Ultrasound	Hypoechoic lesion without vascularity

## Data Availability

Not applicable.

## LIST OF ABBREVIATIONS

CF: Collagenous fibroma
AdCC: Adenoid cystic carcinoma
US: Ultrasonography
FNA: Fine-needle aspiration

## STANDARDS OF REPORTING

CARE guidelines were followed.
